# Case report: Reconstruction exposed bone following excision of malignant scalp tumors, multidisciplinary approach of an old method with new materials

**DOI:** 10.1016/j.ijscr.2023.108484

**Published:** 2023-07-13

**Authors:** Mauro Carducci, Giorgio Butti, Gianluca Nolli, Maurizio Muscarà

**Affiliations:** a Dermatologic Department, Centro Ortopedico di Quadrante, Ospedale Madonna del Popolo, Omegna, Italy; b Neurosurgeon, Orthopedic Department, Centro Ortopedico di Quadrante, Ospedale Madonna del Popolo, Omegna, Italy; c Pathology Department, Castelli Hospital, Verbania, Italy

**Keywords:** Scalp, Squamous cell carcinoma, Reconstruction of the scalp, Dermal matrix

## Abstract

**Introduction:**

Basal cell carcinoma and squamous cell carcinoma are malignant epithelial tumors that frequently occur on the scalp. The reconstruction of large surgical breaches in which the scalp was removed leaving the bone bare has always been a difficult problem to solve.

**Case presentation:**

An 84-year-old patient in good general condition with an extensive surgical breach in the scalp comes to our observation where a sessile squamous cell carcinoma was removed 2 months ago in another hospital; The surgeon had removed the aponeurotic galea with bone exposure.

**Clinical discussion:**

There are numerous surgical solutions proposed: reconstruction by a secondary intention, grafts of dermal matrix, transposition flaps and finally an old technique that involves the perforation of the cranial theca. Dermal matrices cannot be used on bone as they still need blood support to facilitate the repair process. Local flaps could not be used as the skin was seriously photodamaged and treatment of this would further delay the repair. In our case the solutions adopted with other patients were not applicable, therefore we evaluated the literature to determine which solution could be adopted. We had experience with tissue perforation in large ulcers and position punch grafting to facilitate re-epithelialization therefore we also drew inspiration from this method.

**Conclusion:**

The technique adopted allowed an immediate reconstruction limiting the discomfort to the patient with few dressings and complete healing in about 1 month. Scalp, the graft is completely rooted in 3 month.

## Highlights

• Large surgical breaches in the scalp removed leaving the bone bare have been a problem to solve.
• Dermal matrices facilitate the engraftment of full-thickness grafts.
• It is important that surgical procedures limit patient discomfort in the weeks following surgery.

## 1 Introduction

Basal cell carcinoma and squamous cell carcinoma are malignant epithelial tumors that frequently occur on the scalp.

The reconstruction of large surgical breaches in which the scalp was removed leaving the bone bare has always been a difficult problem to solve.

It is essential in dermo-surgery where it is possible to avoid remove the aponeurotic galea which is a fundamental element for using skin grafts.

There are several solutions proposed in the event that the bone remains exposed, we considered the possibility of direct closure, but the surrounding skin was severely photodamaged with numerous actinic lesions and treatment of these would have further delayed closure of the surgical wound.

We preferred to use the drilling technique of which we had similar experience for the reconstruction of extensive ulcers refractory to traditional treatments.

Reconstruction Exposed bone following Excision of Malignant Scalp Tumors, multidisciplinary approach of an old method with new materials.

The patient was managed in a public hospital, a provincial reference center of the Piedmontese oncological network for skin cancer. All services and hospitalization were taken care of by the National Health Service.

Patient operated on in early September 2021 in another hospital for infiltrative squamous cell carcinoma of the scalp.

The surgical beak with exposed bone was left open and after numerous unsuccessful medications, the patient came to our clinic at the end of October.

After 3 days we admit him to the medical department to treat the injury with Vac therapy and prepare him for surgery which is performed after 5 days. The post-surgical hospital course was 1 week (Fig. 7).

Subsequently, the patient was treated in one of our outpatient services until he recovered completely.

## 2 Method and material

An 84-year-old patient in good general condition with an extensive surgical breach in the scalp comes to our observation where a sessile squamous cell carcinoma was removed 2 months ago in another hospital; The surgeon had removed the aponeurotic galea with bone exposure.

The numerous dressings that had been done had achieved to no significant improvements for two months, so much so that the patient turned to our department.

The surrounding part of skin had significant photo damage with actinic lesions, some of which were evolving epitheliomatous.

Histology had confirmed a squamous cell carcinoma of the skin NAS (according to WHO 4th Edition, 2018).

Histologic examination revealed a moderately differentiated (G2) deeply invasive squamous cell carcinoma NOS with infiltrative margins and a tumour thickness >6 mm, without evidence of vascular and perineural invasion, in a background of actinic keratosis; the tumour infiltrated the underlying galea up to the edge of the inked margin. The maximum diameter of the tumour was 55 mm (Fig. 5 and Fig. 6).

**Fig. 1:**
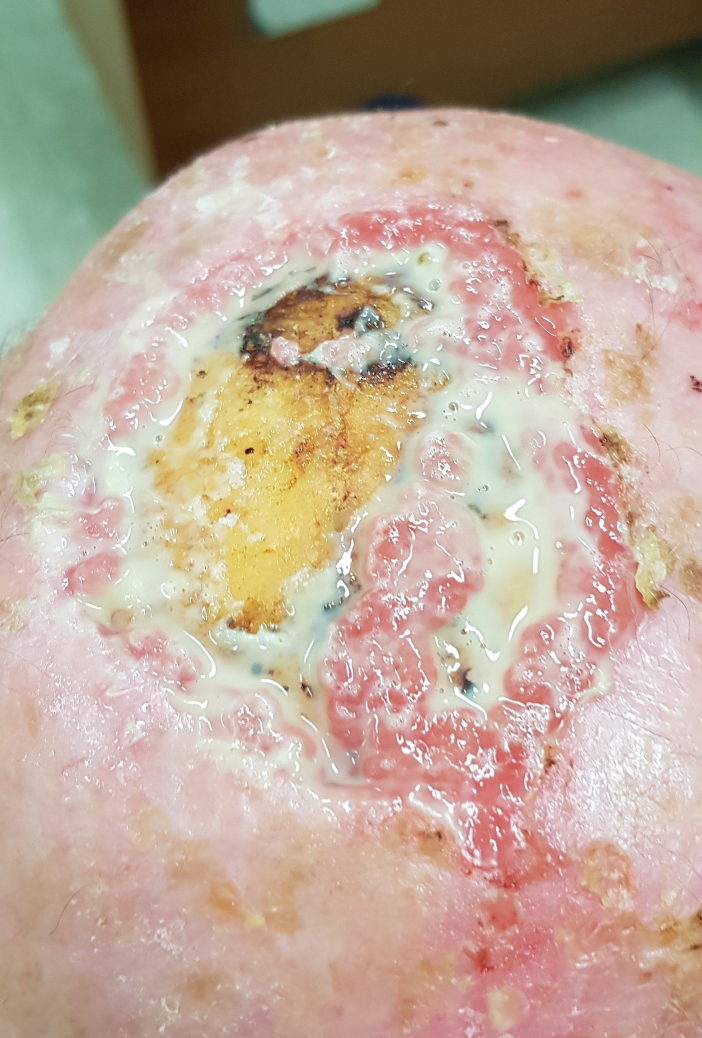
Extensive surgical breach in the scalp comes to our observation where a sessile squamous cell carcinoma was removed 2 months ago.

The wound of about 7 cm in diameter was covered with purulent discharge (Fig. 1).

The patient is hospitalized for intravenous antibiotic therapy and a wound dressing is performed every day with a non-alcoholic disinfectant solution. After removing the necrotic tissue and purulent secretion, it shows the initial suffering of the external cranial table.

After 3 days of antibiotic coverage, an intermittent vacuum therapy at 80 mm hg is placed for one week to clean all the external skull table and stimulating granulation tissue at the periphery of the surgical wound.

The granulation tissue is present only in some minute areas mainly in the periphery.

The procedure was proposed by the dermosurgeon, author of this article, and shared with the anesthetist and neurosurgeon [10].

The surgery is performed under general anesthesia. The surgical team is made up of a neurosurgeon and 2 dermal surgeons. The surgical time from the beginning of the cut to closure was 1 h and 10 min. An intraoperative antibiotic coverage was made with 2 g. of intravenous cefazolin and a post-surgical antibiotic coverage with 1 g of ampicillin and clavulanic acid every 8 h for 10 days.

**Fig. 2:**
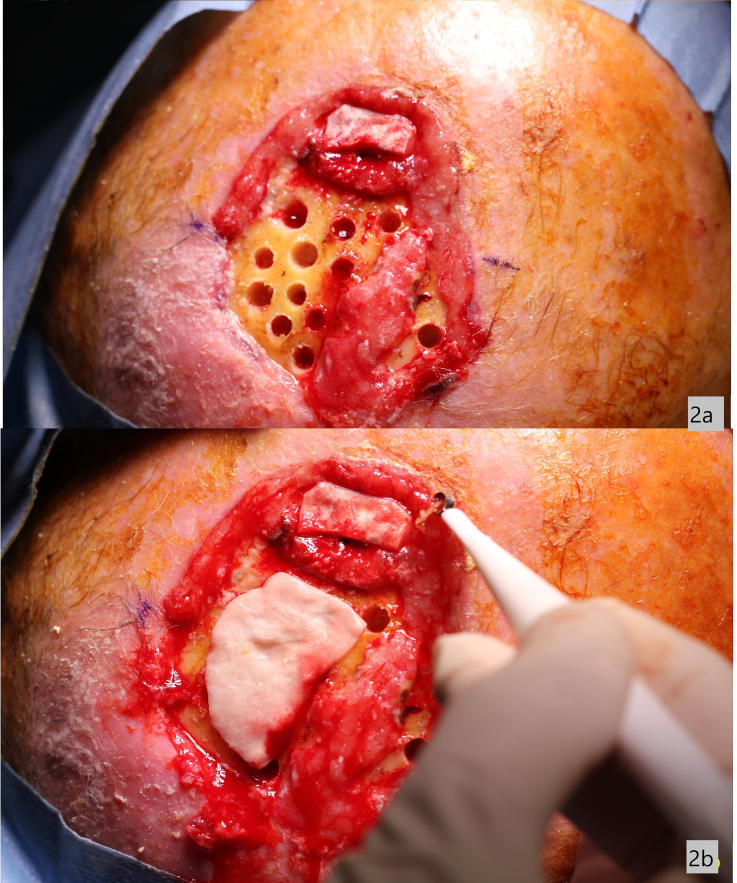
a) Approximately 15 holes of 4–5 mm in diameter are drilled using a high-speed cutting bur and subsequently a curettage to bloody the diploe to facilitate bleeding. b) A dermal matrix in spongy plaques of hyaluronic acid, native type I equine collagen is placed on bone tissue with bleeding breccias.

Approximately 15 holes of 4–5 mm in diameter are drilled using a high-speed cutting bur and subsequently a curettage to bloody the diploe to facilitate bleeding (Fig. 2).

The holes were drilled taking care not to injure the dura. The perforation was stopped when bleeding from the diploic spaces occurred.

A dermal matrix Type 1 heterologous equine collagen and hyaluronic acid in sterile sponge plates 10 × 10 cm adaptable to the site, thickness about 2 mm is placed on bone tissue with bleeding breccias.

**Fig. 3:**
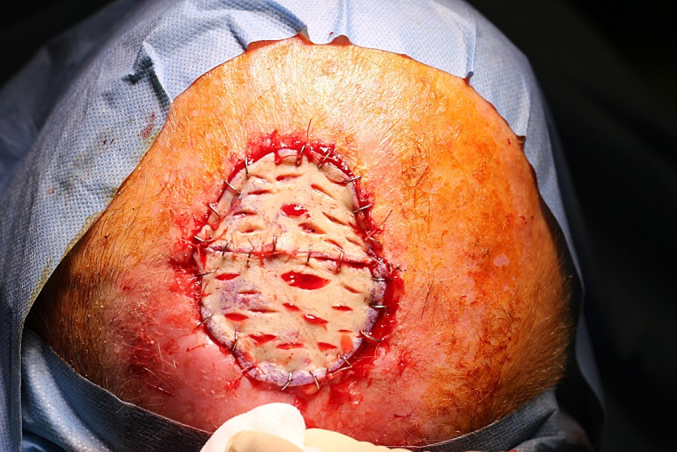
The graft divided into 2 fragments removed from the thigh, degreased and thinned, positioned in the breach and fixed with staples and resorbable 3/0 surgical threads.

The graft divided into 2 fragments removed from the thigh, degreased and thinned, positioned in the breach and fixed with staples and resorbable 3/0 surgical threads. The graft is fenestrated and medicated with greasy gauze and a moderate compression dressing (Fig. 3).

Bleeding was limited and controlled with diathermocoagulation, it was not necessary to proceed with blood transfusions.

The dressing is removed after 8 days and the stitches after 12 days.

The patient was checked every week for the first month then every 2 weeks for 3 months.

**Fig. 4:**
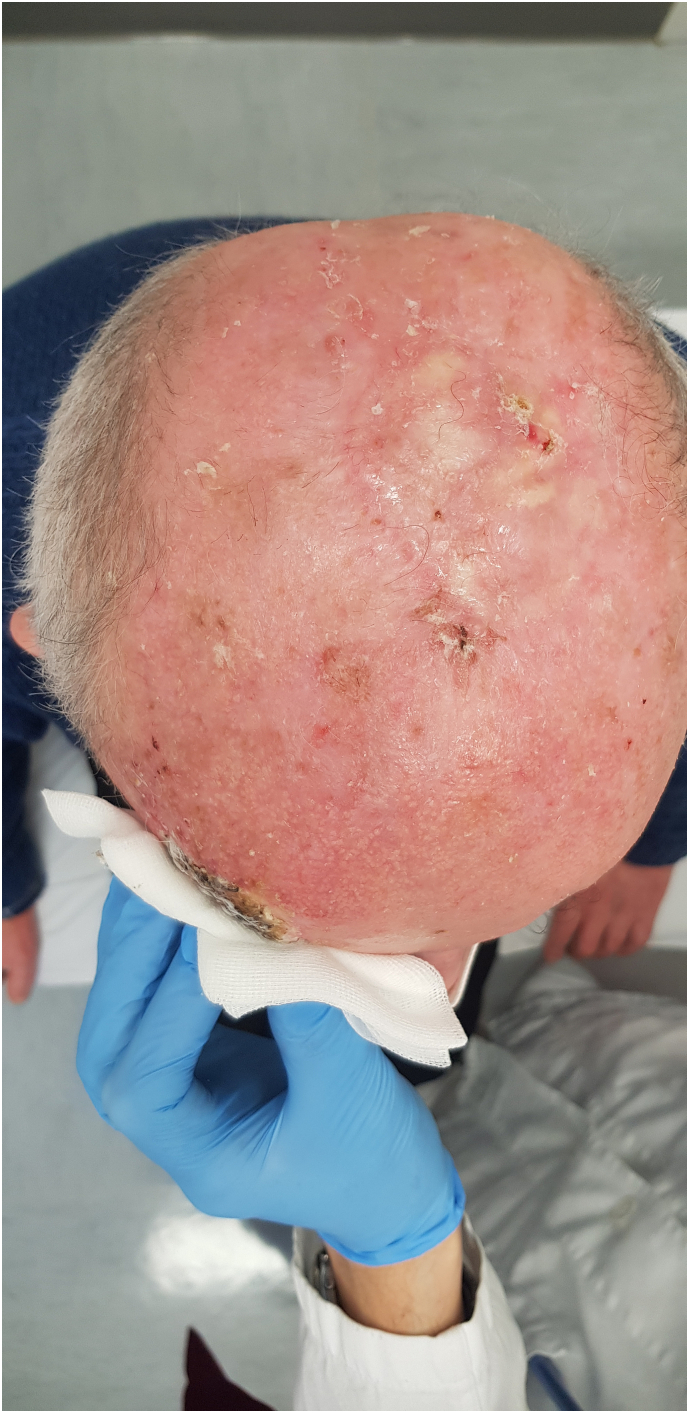
Scalp, the graft is completely rooted in 3 months. Control visit at 6 months.

The graft is completely rooted (Fig. 4).

The work has been reported in line with the SCARE criteria.

## 3 Discussion

The reconstruction of the scalp, with the exposure of the bone, has always been a difficult problem to solve. There are numerous surgical solutions proposed: reconstruction by a secondary intention [1], grafts of dermal matrices [2], transposition flaps [3] and finally an old technique that involves the perforation of the cranial theca [4].

Secondary intention reconstruction takes a very long time and creates considerable inconvenience to the patient.

The use of dermal matrices is similar to the previous solution with perhaps shorter times [5].

Thin grafts are only a temporary solution and in any case it is necessary to create a blood supply condition.

The milling of the cortical theca reduces the solidity of the theca itself, therefore we have identified in this old method a good solution with the variant of inserting a dermal matrix to immediately rebuild with a medium thickness graft (Fig. 2).

The presence of a tissue surrounding the surgical breach with numerous actinic lesions, some of which are in epitheliomatous evolution, excluded the possibility of practising transposition in the flap from neighburing areas with grafting in the site of donation of the flap.

In the history of plastic surgery we find references of this technique dating back to the 18th century. The earliest reference we have found of this technique came from a study by Mellish [6] in 1904 who mentioned that a doctor in 1777 who ordered a colleague to puncture the skull of a patient with scalp loss until a reddish fluid came out which aided the formation of granulation. The skin on the sides has grown very slowly over time.

In 1914 Flaherty [7] described the technique in more detail using a drill to make holes in the outer table of the skull and then proceeded to graft once the granulation tissue was formed.

Over time, rotational flaps and microvascular flaps have become the gold standard for reconstruction of surgical breccias with an exposed skull table. Their main advantage was the lack of waiting time for wound granulation and the elimination of the need for repeated dressings.

Furlanetti et al. [8] Mentioned that the technique of multiple perforation with subsequent grafting which can also be used in total avulsion of the scalp when microsurgical reimplantation fails or is not feasible because the skull is broadly exposed.

Calderoni et al. [9] also stated that the procedure helps to form granulation tissue by associating the use of skin expanders that can be used in adjacent sites to increase the tissue available for closure.

It was not easy to find a solution that would lead to a quick resolution of the problem that had been going on for months.

Dermal matrices cannot be used on bone as they still need blood support to facilitate the repair process. Local flaps could not be used as the skin was seriously photodamaged and treatment of this would further delay the repair. In our case the solutions adopted with other patients were not applicable, therefore we evaluated the literature to determine which solution could be adopted. We had experience with tissue perforation in large ulcers and position punch grafting to facilitate re-epithelialization therefore we also drew inspiration from this method.

Certainly the infectious risk in a technique such as the one adopted is possible. We must consider that the wound arrived covered in purulent material and that the brief pre-surgical treatment healed it.

## 4 Conclusion

The technique adopted allowed an immediate reconstruction limiting the discomfort to the patient with few dressings and complete healing in about 1 month. Scalp, the graft is completely rooted in 3 months. Control visit at 6 months (Fig. 7).

**Fig. 5:**
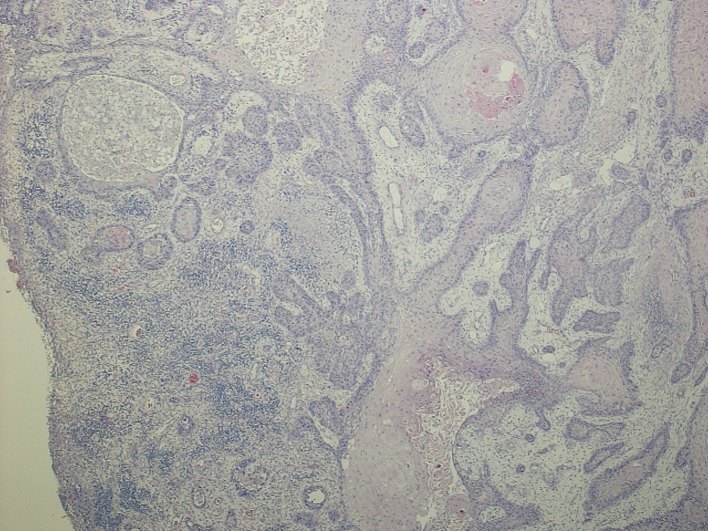
Scanning magnification (4×) shows a moderately differentiated SCC with infiltrative growth pattern.

Minimally invasive surgery with reduced surgical times that does not identify significant contraindications other than those related to general anesthesia (Fig. 5, Fig. 7).

**Fig. 6:**
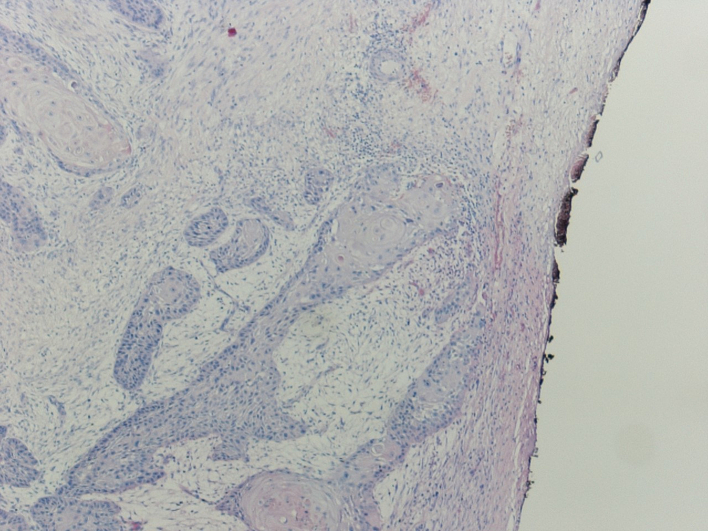
The tumour infiltrates the galea up close the resection margin (original magnification 10×).

**Fig. 7:**
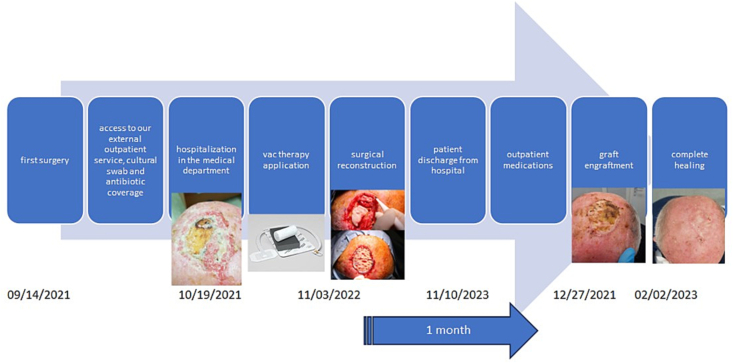
A graphical/visual representation of timeline of events.

The patient was managed in a public hospital, a provincial reference center of the Piedmontese oncological network for skin cancer. All services and hospitalization were taken care of by the National Health Service.

## Consent

Written informed consent was obtained from the patient for publication of this case report and accompanying images. A copy of the written consent is available for review by the Editor-in-Chief of this journal on request.

## Ethical approval

Access to the ethics committee and its authorizations are not applicable in our case because the individual surgical and therapeutic techniques are consolidated and do not represent a study.

## Funding

No any sources of funding for your research.

## Author contribution

Each author contributed study concept and design, data collection, data analysis or interpretation, writing the paper.

## Guarantor

Mauro Carducci, MD.

## Research registration number

The ethics committee clearance was not necessary for e standard surgical treatment. Not applicable in our case because the individual surgical and therapeutic techniques used, are consolidated and do not represent a study of research.

## Declaration of competing interest

All the authors declare that they have no conflicts of interest.

## References

[1] Becker G.D., Adams L.A., Levin B.C. (1999). Secondary intention healing of exposed scalp and forehead bone after mohs surgery. Otolaryngol. Head Neck Surg..

[2] Magnoni C., De Santis G., Fraccalvieri M., Bellini P. (Nov-Dec 2019). Integra in scalp reconstruction after tumor excision: recommendations from a multidisciplinary advisory board. J. Craniofac. Surg..

[3] Zhou Y., Jiang Z., Li C., Cai Y., Sun R., Shui C. (2020). An algorithm for one-stage malignant oncologic scalp reconstruction. Ann. Transl. Med..

[4] Chatterjee D., Ghosh N., Patel S.M., Krishnan P. (2019). Exposed bone with scalp and pericranial loss: role of multiple calvarial drillings in aiding closure. J. Pediatr. Neurosci..

[5] Chun Y.S., Verma K. (2011). Single-stage full-thickness scalp reconstruction using acellular dermal matrix and skin graft. Eplasty.

[6] Mellish E.J. (Nov 1904). Total avulsion of the scalp. Ann. Surg..

[7] Flaherty F. (1914). Complete avulsion of the scalp. With a report of a case. Ann. Surg..

[8] Furlanetti L.L., de Oliveira R.S., Santos M.V., Farina J.A., Machado H.R. (Jun 2010). Multiple cranial burr holes as an alternative treatment for total scalp avulsion. Childs Nerv. Syst..

[9] Calderoni D., Rosim E., Kharmandayan P. (2012). Successful calvarial bone salvage using multiple outer table perforation technique on total scalp avulsion injury. Eur. J. Plast. Surg..

[10] Agha R.A., Franchi T., Sohrab C., Mathew G., Kirwan A., Thomas A. (2020). The SCARE 2020 guideline: updating consensus Surgical Case Report (SCARE) guidelines. Int. J. Surg..

